# Prevention of Hypertensive Disorders of Pregnancy—Is There a Place for Metformin?

**DOI:** 10.3390/jcm10132805

**Published:** 2021-06-25

**Authors:** Elżbieta Poniedziałek-Czajkowska, Radzisław Mierzyński, Dominik Dłuski, Bożena Leszczyńska-Gorzelak

**Affiliations:** Chair and Department of Obstetrics and Perinatology, Medical University of Lublin, 20-059 Lublin, Poland; radek@bg.umlub.pl (R.M.); p.l.casiraghi@wp.pl (D.D.); b.leszczynska@umlub.pl (B.L.-G.)

**Keywords:** preeclampsia, pregnancy-induced hypertension, metformin, pregnancy

## Abstract

The possibility of prophylaxis of hypertensive disorders of pregnancy (HDPs) such as preeclampsia (PE) and pregnancy-induced hypertension is of interest due to the unpredictable course of these diseases and the risks they carry for both mother and fetus. It has been proven that their development is associated with the presence of the placenta, and the processes that initiate it begin at the time of the abnormal invasion of the trophoblast in early pregnancy. The ideal HDP prophylaxis should alleviate the influence of risk factors and, at the same time, promote physiological trophoblast invasion and maintain the physiologic endothelium function without any harm to both mother and fetus. So far, aspirin is the only effective and recommended pharmacological agent for the prevention of HDPs in high-risk groups. Metformin is a hypoglycemic drug with a proven protective effect on the cardiovascular system. Respecting the anti-inflammatory properties of metformin and its favorable impact on the endothelium, it seems to be an interesting option for HDP prophylaxis. The results of previous studies on such use of metformin are ambiguous, although they indicate that in a certain group of pregnant women, it might be effective in preventing hypertensive complications. The aim of this study is to present the possibility of metformin in the prevention of hypertensive disorders of pregnancy with respect to its impact on the pathogenic elements of development

## 1. Introduction

The frequency of hypertensive diseases is estimated at 8–10% of pregnancies: chronic hypertension is observed in 0.9–1.5% of pregnant women and pregnancy-induced hypertension in 2–8% [1,2]. Preeclampsia is diagnosed in 1% of pregnant women and 1.5% of primiparas [3]. According to WHO (World Health Organisation) reports, it is responsible for about 76,000 maternal deaths per year, which accounts for 16% of all maternal deaths, primarily in developing countries. In developed countries, it is the most common cause of iatrogenic premature births [4,5].

Preeclampsia is a complex of clinical symptoms that occur after the 20th week of pregnancy. According to the classification of the American College of Obstetricians and Gynecologists (ACOG), preeclampsia (PE) is defined by new-onset hypertension after the 20th week of gestation, with systolic blood pressure ≥140 mmHg or diastolic blood pressure ≥90 mmHg, measured on two occasions at least four hours apart, and proteinuria of ≥0.3 g per 24 h or ≥1+ proteinuria, detected by urine dipstick. In the absence of proteinuria, preeclampsia is defined by new-onset hypertension with new onset of any of the following: thrombocytopenia (platelet count <100,000/μL), renal insufficiency (serum creatinine concentration >1.1 mg/dL or a doubling of serum creatinine concentration in the absence of other renal diseases), impaired liver function (raised concentrations of liver transaminases to twice normal concentrations), pulmonary edema, or cerebral or visual problems [2].

Another form of hypertension that occurs in pregnancy is gestational hypertension (GH) or pregnancy-induced hypertension (PIH), with systolic blood pressure ≥140 mmHg or diastolic blood pressure ≥90 mmHg, that is diagnosed after the 20th week in a previously healthy woman, with no signs of proteinuria or other symptoms characteristic of preeclampsia [2]. 

Pregnancy-induced hypertension can result in an adverse pregnancy outcome as well as precede PE development; hence, the management of PIH is similar to that of preeclampsia. At present, the only effective, causal form of PE treatment is delivery. The prevention of the development of hypertension in pregnancy, mainly PE, is extremely important due to its unpredictable course and the risk of unfavorable outcomes for both mother and fetus or newborn. The ideal PE prevention should alleviate the influence of risk factors and, at the same time, promote physiological trophoblast invasion and maintain the physiologic endothelium function without any harm to both mother and fetus. Effective and safe prophylactic methods have remained of great interest for many years. So far, aspirin is the only drug with recognized efficacy in PE prevention, recommended by many institutions around the world for use in patients in high-risk groups [2,6,7,8].

The results of the latest studies on preeclampsia pathogenesis and its better understanding justify research on the possibility of using other drugs for prevention. There are reports of the use of sulfasalazine, folate, nitric oxide donors (such as L-Arginine), antithrombin III, and antioxidants such as melatonin [9].

Metformin, a pharmacological hypoglycemic agent, has been known for decades [10,11]. At present, metformin is called aspirin of the 21st century due to its other than only hypoglycemic properties, such as anticancer, antiaging, protective effect on the endothelium and prevention of cardiovascular events, beneficial effects on body weight, and lipid profile [12].

Preeclampsia is a syndrome involving the vasculature of different organs; it can be found as a form of cardiovascular disease affecting, predominantly, the trophoblast/placenta, where abnormal invasion and function is a starting point to initiate PE development. It has been proven that of all hypoglycemic drugs, only metformin has a beneficial effect on the circulatory system, as underlined by the American Association of Clinical Endocrinologists (AACE). This institution recommends its use as an agent of first choice in patients with type 2 diabetes mellitus (DMt2) [10].

The purpose of this study is to present the possibility of metformin in the prevention of PE and PIH with respect to its impact on the pathogenic elements of development.

## 2. Pathophysiology of Preeclampsia

The pathogenesis and pathophysiology of PE are still poorly or incompletely understood. The main pathological feature of early-onset PE is the incomplete transformation of the spiral arteries, resulting in the hypoperfusion of the placenta and reduced transfer to the fetus. In this type of PE, a significantly higher risk of maternal and fetal complications, such as fetal growth restriction (FGR), is observed [13]. It has been proposed that preeclampsia be divided into two main categories: early-onset (<34 weeks of gestation) and late-onset (>34 weeks of gestation). These two subtypes seem to have different pathophysiological and etiological pathways [14].

In late-onset preeclampsia, the spiral arteries are slightly changed in diameter; the cause arises from the interaction between a probably normal placenta and maternal factors which are connected with endothelial dysfunction. Maternal late-onset preeclampsia is associated with a lower rate of fetal complications [15].

According to the hypothesis of insufficient trophoblastic invasion with associated uteroplacental hypoperfusion, a two-stage model of PE was proposed: incomplete spiral artery remodeling in the uterus that contributes to placental ischemia (Stage 1) and the release of antiangiogenic factors from the ischemic placenta into maternal circulation that results in endothelial damage (Stage 2) [13]. During implantation, the placental trophoblast invades the uterus and induces the remodeling of the spiral arteries while obliterating the tunica media of the myometrial spiral arteries. This enables the arteries to accommodate increased blood flow regardless of the maternal vasomotor changes [16]. Thus, the major impact of the remodeling of the spiral arteries is to increase the perfusion of the intervillous space [15]. If this process fails, the placenta is likely to be deprived of oxygen, leading to a state of relative ischemia and an enhancement in oxidative stress [16].

The key issue remains the cause of abnormal trophoblast implantation. Many researchers have suggested that it is an impaired response of the maternal immune system or an abnormal tolerance to the development of the allogenic fetus [17,18]. Several studies have been conducted on immunological changes in pregnancies complicated by preeclampsia. They have indicated the excessive activation of neutrophils and monocytes, which synthesize large amounts of proinflammatory cytokines such as IL-1β, IL-6, and IL-8 [19,20]. In addition, CD4+ and CD8+ T-cells, together with NK (natural killer) and dendritic cells (DCs), show a different response in PE women compared to healthy pregnant women. In physiological pregnancy, a dominance of anti-inflammatory response and immunosuppression is observed [21]. Decidual NK cells (dNKcs), which are 70% of the inflammatory cells present in the decidua, play an important role in proper trophoblast implantation and the remodeling of spiral arteries [22]. The potential role of dNKcs was proposed by studies revealing that if dNKcs are knocked out in genetically modified laboratory animals, physiological spiral artery remodeling is not observed [23]. 

Impaired remodeling of spiral arteries, reduced placental blood flow, and oxidative and endoplasmic reticulum stress may have both systemic and local effects. The placental alterations associated with poor remodeling of the uteroplacental spiral arteries lead to the release of several placental factors into maternal circulation that contributes to the development of increased inflammatory response, oxidative stress, apoptosis, the release of syncytiotrophoblast microparticles, and endothelial dysfunction [24]. 

A healthy endothelium possesses autocrine, paracrine, and endocrine properties and produces a variety of vasoactive mediators. Nitric oxide (NO), prostacyclin I2 (PGI2), endothelium-derived hyperpolarizing factor (EDHF), bradykinin, histamine, serotonin, and substance P are the main vasodilators synthesized within endothelial cells. Endothelin-1 (ET-1), angiotensin II (ANG-II), thromboxane A2, prostacyclin H2, and reactive oxygen species (ROS) represent vasoconstrictors. The functional stability of endothelium is assured by the balance of secreted vasoconstrictors and vasodilators; the asymmetry in their synthesis is responsible for many pathophysiological changes, including preeclampsia. The impaired function of the endothelium is determined by at least one of the changes, such as a decrease in NO synthesis and bioavailability, higher adhesion molecule and inflammatory gene expression, intensified ROS synthesis, impaired endothelium-dependent vasorelaxation, decreased fibrinolysis, and enhanced endothelial permeability [25].

Disturbed endothelial function is considered one of the most characteristic features of PE that leads to generalized vasoconstriction and restricted organ perfusion. Pre-existing risk factors such as obesity, diabetes, and poor nutrition adversely affect endothelial function and, finally, exacerbate maternal response to signals from the hypoxic placenta [26]. The results of the studies presented by Zhao et al. have suggested a more significant deterioration of endothelial function in the course of PE compared to PIH, which may partly explain the more severe clinical course of preeclampsia [27]. 

It has been believed that angiogenic and antiangiogenic factors produced by the placenta are influenced by hypoxia and oxidative stress and play a crucial role in the pathophysiological features of preeclampsia [28]. Vascular endothelial growth factor (VEGF) and placental growth factor (PlGF) play a key role in placental angiogenesis in physiological and pathological pregnancy. VEGF exerts its effects via the binding and activation of two cell surface receptor tyrosine kinases, VEGFR-1/Flt-1 and VEGFR-2/KDR (vascular endothelial growth factor receptor), that are presented on endothelial cells [29]. VEGF has been described as a potent stimulator of endothelial cell proliferation and synthesis of plasminogen activators. Both of these mechanisms are markers of angiogenic activity [30]. VEGF is essential for the integrity of endothelial cells [31]. A link between VEGF and oxidative stress in the placenta has been postulated. Koroglu et al. have suggested that changes in VEGF levels may increase 5′ adenosine monophosphate-activated protein kinase (AMPK) activity in patients with severe preeclampsia due to placental hypoperfusion [32]. 

AMPK is a heterotrimeric serine–threonine protein kinase that is expressed in almost every cell type and tissue within the body [33]. AMPK plays a role in several processes, such as oxygen regulation, cellular energy homeostasis, and metabolism as well as placental angiogenesis [34]. It is known that AMPK is present in the human placenta; its levels increase under hypoxic conditions, as seen in preeclampsia, and maintain the appropriate blood flow in maternal uterine arteries [35]. In the study presented by Koroglu et al., it was concluded that higher levels of AMPK revealed in severe PE may be a compensatory mechanism to balance angiogenic and antiangiogenic molecules that induce endothelial dysfunction [32]. PlGF is an angiogenic protein belonging to a vascular endothelial growth factor family and has 53% homology with VEGF. PIGF influences endothelial cell adhesion and chemotaxis and appears to increase the angiogenic effect of VEGF [36,37]. Like VEGF, it binds to the sFlt-1 receptor, resulting in non-branching angiogenesis [38]. 

The main antiangiogenic factors that play a role in the pathogenesis of preeclampsia are VEGF receptors (VEGFR1 and VEGFR2) and soluble endoglin (sEng). VEGFR1 is also known as fms-like tyrosine kinase-1 (sFlt-1) [39]. sFlt-1 secretion is regulated through the mitochondria. Inhibiting the mitochondrial electron transport chain reduces sFlt-1 secretion from primary villous cytotrophoblasts cells. It has been proven that mitochondrial electron transport chain activity in preterm preeclamptic patients is increased compared to patients with uncomplicated pregnancies [40]. sEng is known to be an extracellular domain of full-length membrane endoglin, and it decreases its proangiogenic and vasodilators impact [41]. Elevated concentrations of sEng have been described in the serum of preeclamptic patients [42]. 

Preeclampsia is associated with placental ischemia/hypoxia and an increase in the secretion of sFlT-1 and sEng into maternal circulation [43]. This causes widespread endothelial dysfunction that manifests clinically as hypertension and multisystem organ injury [44]. It has been suggested that an imbalance between angiogenic and antiangiogenic factors influences the pathophysiological changes observed in PE, and it appears before clinical symptoms are noticed [45]. It has been described that the circulating level of PlGF is lower in patients who will be preeclamptic before the increase in s-Flt [46]. In the study published by Maynard et al., it was revealed that at the time of delivery, sFlt-1 was upregulated in the circulation of preeclamptic patients; decreased VEGF and PIGF levels were also observed. When sFlt-1 was administered to rodents via an adenovirus, they developed marked hypertension and albuminuria and histologic changes characteristic of PE (i.e., glomerular enlargement, endotheliosis, and fibrin deposition within the glomeruli). sFlt-1 is supposed to be the main mediator in the development of PE [47]. Govender et al. noticed that serum sFlt-1 levels were significantly higher in early-onset PE and higher in late-onset PE compared to normotensive patients and chronic hypertensive patients, while VEGF was not detectable in all groups [48]. Venkatesha et al. found that both sEng and sFlt-1 could block the actions of VEGF [42]. By this mechanism, sEng may decrease the activation of endothelial nitric oxide synthase (eNOS) and the synthesis of the potent vasodilator nitric oxide, which is crucial for appropriate trophoblast development and invasion [49]. NO bioavailability is positively correlated with VEGF and PIGF, which enhance endothelial NO synthesis [50,51]. Thus, the impaired balance of angiogenic and antiangiogenic factors observed in PE influences NO synthesis. sFlt-1 decreases the availability of free PlGF and VEGF and, finally, leads to the decline in NO synthesis, which is also disturbed by oxidative stress and ROS involved in PE pathogenesis [52,53].

sEng is believed to be one of the potent antiangiogenic factors, the levels of which are raised in preeclampsia [54]. It has been observed that the administration of sEng to pregnant rodents markedly increased blood pressure at the 17th to 18th day of pregnancy but led only to mild-to-modest proteinuria while sFlt-1 infusion resulted in high levels of proteinuria, hypertension, and features of HELLP syndrome [42]. It has been suggested that the increased secretion of sFlt-1 and sEng into maternal circulation could be responsible for widespread endothelial dysfunction that manifests clinically as hypertension and multiorgan injury [44].

It has been observed that hypoxia-inducible factor 1α (HIF1α), the central mediator of hypoxic response, plays an important role in placental development and function. It is upregulated with ischemia/hypoxia. HIF-1α has been demonstrated to induce the production of sFlt-1 in placental explants [55]. A growing body of evidence supports HIF-1α being the molecular link between placental hypoxia and the downstream mediators of preeclampsia. Therefore, drugs that can block HIF1α activity may decrease sFlt-1 secretion [56]. It has also been postulated that some pharmacologic agents that are safe in pregnancy, by reducing placental sFlt-1 and sENG synthesis may improve endothelial dysfunction and, finally, may be effective in preventing and treating preeclampsia [40].

Endoplasmic reticulum stress (ERS), which may be the result of factors such as abnormal glucose metabolism, viral infection, or oxidative stress, is believed to be involved in the pathogenesis of preeclampsia. It promotes the release of proinflammatory cytokines, antiangiogenic factors, and trophoblastic apoptotic debris, and all of them have been found to diminish endothelial function [57,58,59]. Fu et al. observed that ERS-induced apoptosis was important in the development of severe PE, especially in its early onset [60].

Placentation involves extracellular matrix degradation with matrix metalloproteinases (MMPs), the expression/activity of which is augmented in normal pregnancy. The invasive potential of extravillous trophoblast cells relates to MMP-2 and MMP-9 expression [61]. The decreased vascular MMP-2 and MMP-9 expression enhance the vasoconstriction observed in preeclampsia. In animal models, antiangiogenic factors such as sFlt-1 have been observed to diminish MMP expression/activity within placental tissue and vascular walls, while angiogenic factors such as VEGF have been found to transpose this process and to improve placentation [62].

Vascular integrity is an essential feature of the physiological endothelium. Endothelial glycocalyx, one of the matrix structures, prevents increase in endothelial permeability. Endothelial dysfunction, characteristic of PE, can lead to increased vascular permeability, as can endothelial glycocalix disorder [63]. Vascular permeability was more than five times higher in preeclamptic patients than in healthy pregnant women [64]. It has been suggested that an imbalance between angiogenic and antiangiogenic factors may be responsible for increased vascular permeability [47].

Endothelial progenitor cells (EPCs) are endothelial cell precursors that can enhance endothelial repair, influence the remodeling of vessels, and improve angiogenesis [65]. King et al. observed that mothers of small-for-gestational-age infants had a reduced number of EPCs, with limited migration function. It may support the hypothesis of the adverse effects of EPC disturbances on placentation, which may contribute to an increased risk of maternal cardiovascular complications [66]. Actually, in preeclamptic patients, significantly lower EPCs have been noticed compared to healthy patients [67,68]. The decrease in EPCs number and their functional impairment in patients with PE might be associated with endothelial dysfunction. It has been postulated that it could be the result of systematic inflammation and the influence of increased sFlt-1 levels [68,69]. Matsubara et al. concluded that although certain factors such as TNF-alpha or angiotensin II stimulate the proliferation of EPCs, their incomplete release into the circulation prevents the renewal of endothelium cells in preeclampsia [70]. The transfer of EPCs from healthy pregnant rats to PE rats significantly improved placental perfusion [71].

Sirtuin 1 (SIRT1) overexpression is believed to significantly improve cell survival, reduce apoptosis, and also reduce the release of proinflammatory cytokines [72]. Only a few studies on SIRT1 in HDPs pathogenesis are available. Reduced SIRT1 expression has been demonstrated in PE patients compared to PIH patients and healthy pregnant women, which may suggest a different pathogenetic background of PIH and PE [73]. Its decreased levels have been noticed in third-trimester placentas from patients with PE [74]. Yin et al. revealed a reduction in the concentration of this protein in human umbilical endothelial cells incubated with serum from PE patients. The authors concluded that SIRT1 may have a protective effect on the placenta in PE [75].

The real significance of angiogenic and antiangiogenic factors, however, may depend on their ability to predict maternal and fetal/neonatal complications. In patients with the clinical diagnosis of preeclampsia, it has been demonstrated that increased sFlt/PlGF ratios are correlated with worse maternal and fetal outcomes compared to women with lower ratios. In patients with suspected early-onset preeclampsia, the circulating sFlt1/PlGF ratio can predict adverse outcomes that will occur within two weeks [76]. In 2016, the multicenter study that analyzed high-risk pregnant women in their second and third trimesters using angiogenic markers was published. It shows that an sFlt-1/PlGF ratio of 38 or lower, assessed at 24–37 weeks of gestation, can reliably predict the absence of PE and adverse fetal outcomes within 1 week, with negative predictive values of more than 99% [77].

Abnormal trophoblast implantation underlying the development of PE is responsible for the subsequent restriction of blood flow in uteroplacental circulation. An increase in resistance within this circulation can be diagnosed with uterine artery Doppler flow velocimetry between 11 + 0–13 + 6th week using the pulsation index (PI) in both uterine arteries. Restricted placental perfusion is reflected in the increase in mean PI values and the subsequent development of preeclampsia [78]. The Fetal Medicine Foundation has proposed an early preeclampsia prediction algorithm using maternal data, PI values in uterine arteries, and concentrations of PIGF and PAPP-A (pregnancy-associated plasma protein A). The risk, estimated at more than 1:100, indicates the need for prevention with aspirin. Screening based on maternal factors, including mean arterial pressure (MAP), PI values, and PAPP-A and PlGF concentrations, allows to identify 95% of cases of early-onset [79,80].

In the clinical prediction of PE development, risk factors are commonly used, including, but not limited to, the presence of chronic conditions (diabetes mellitus t.1 and t.2, obesity, chronic hypertension, systemic lupus, antiphospholipid syndrome, chronic kidney diseases, PE in the past), as well as multiple pregnancy, advanced maternal age, or first pregnancy. The presence of risk factors, according to recommendations of various gynecological and obstetric societies, is an indication for the prevention of preeclampsia. Currently, the only recommended drug is aspirin at a dose of 75–150 mg per day, which should be offered at the latest by the 16th week of pregnancy [2,6,7,8].

## 3. Metformin

### 3.1. Pharmacokinetics and Mechanism of Action

Metformin is believed to be a safe medicine in pregnancy and is used primarily to treat women with gestational diabetes mellitus (GDM) [81]. Metformin, a dimethyl-biguanide hydrochloride, is an oral hypoglycemic agent of a molecular weight of 129 daltons, absorbed within the duodenum and jejunum and excreted unchanged with urine and bile [82]. The hypoglycemic effect of metformin is the result of several mechanisms, such as reduced gluconeogenesis in the liver, with limited hepatic glucose synthesis; decreased glucose absorption in the gastrointestinal tract; and intensification of its uptake in skeletal muscles [83]. Its maximum dose is estimated to be 2.5–3 g daily (35–42 mg/kg) [84]. It has been found that metformin acts within mitochondria, where it inhibits complex I of the mitochondrial electron transport chain (ETC), which leads to a reduction in nicotinamide adenine dinucleotide (NADH) oxidation and adenosine triphosphate (ATP) synthesis. It results in the activation of the 5′-adenosine monophosphate (AMP) kinase (AMPK), an increase in AMP concentrations, and the inhibition of the cAMP/PKA pathway (protein kinase A). Metformin activates the AMPK pathway, which results in a decrease in hepatic glucose synthesis and an increase in glucose consumption in muscles [85,86]. Additionally, metformin inhibits the transmembrane protein vATPase on the surface of the lysosome and raises the AMP/ATP ratio. It also stimulates AXIN-LKB1-vATPase and enhances AMPK protein attachment to the lysosome surface [85]. The increased AMP/ATP ratio is responsible for the activation of AMPK, which results in suppressed glucose synthesis in the liver, enhanced insulin sensitivity and glucose uptake by muscle, and the activation of fatty acid oxidation [87]. This process demands threonine 172 phosphorylation with liver kinase B1 (LKB1) [88]. The AMPK pathway is triggered by metformin in a dose- and time-dependent manner. However, it has also been postulated that metformin can stimulate AMPK separately from AMP/ATP ratio variations [89].

The results of animal studies have indicated that metformin may also decrease liver glucose synthesis concentrations without AMPK pathway involvement [90]. According to the observations presented by Madiraju et al., gluconeogenesis can be restrained by metformin through mitochondrial glycerophosphate dehydrogenase suppression [91]. AMPK activation influences cellular metabolism and cell growth and proliferation by blocking mTORC (mammalian target of rapamycin), which is a cancer-supporting pathway [92]. Metformin may develop an antiproliferative effect by inhibiting mTORC1 through AMPK [93,94,95].

The underlying mechanism by which metformin reduces the incidence of cardiovascular events and all-cause mortality has been actively investigated. It has been found that the induced AMPK pathway may act not only as a hypoglycemic and antiproliferative agent but also favorably influences the cardiovascular system. It may reduce inflammatory cell proliferation and their adhesion to the endothelium and lipid accumulation, and it is involved in the activation of genes responsible for cellular antioxidant defense and enzymes committed in the synthesis of nitric oxide [96]. As a result, this translates into favorable effects on clinical signs and symptoms, such as hypertension, obesity and overweight, atherogenic dyslipidemia, procoagulant and thrombosis conditions, and carotid intima-media thickness, all of which are improved [97,98]. Metformin has been found to protect against cardiovascular complications, mainly by improving the function of the endothelium and by its anti-inflammatory properties, which have a positive impact on blood pressure, coagulation processes, and overweight/obesity [99,100].

Studies on animal models have proven that metformin can restore normal endothelium function [101].

a. Inflammation and oxidative stress

There are many available reports on in vitro and animal models confirming the antiinflammatory properties of metformin. It has been suggested that metformin, by AMPK pathway activation, can restrain nuclear factor kappa B (NF-κB), which results in the limitation of proinflammatory gene expression [102,103]. NF-κB inhibition by metformin is also the effect of the blockade of the phosphoinositide 3-kinase (PI3K)-Akt pathway in human vascular wall cells [104].

It has been observed that NF-κB suppression in macrophages, monocytes, and lymphocytes may finally result in a decrease in proinflammatory cytokines levels such as IL-1β, IL-6, and tumor necrosis factor-α (TNF-α), monocyte chemoattractant protein-1 (MCP-1), and IL-8, IL-2, and interferon as well as NO and prostaglandin E2 (PGE2) release [105]. Gongol et al. found that metformin may inhibit the TNF-α–induced gene expression regulating E-selectin, vascular cell adhesion molecule 1 (VCAM1), intracellular adhesion molecule 1 (ICAM1), and MCP1 release. All of them contribute to monocyte adhesion to activated endothelial cells, suggesting that metformin could be a useful agent in preventing monocyte adhesion to endothelial cells [106]. Thus, the influence on the NF-κB pathway may represent an interesting target for anti-inflammatory therapies. In addition, metformin has been shown to reduce the proinflammatory response by affecting AMPK-phosphatase and the tensin homolog (PTEN) [107].

Metformin also diminishes the synthesis of advanced glycation end-products (AGEs), the levels of which increase due to hyperglycemia. AGEs have been found to exert proinflammatory properties and are believed to be one of the reasons for the development of vascular complications in diabetes mellitus [108,109]. AGEs have been revealed to induce oxidative stress as well as activate proinflammatory processes in the endothelium [110]. Mamptu et al. observed that metformin inhibits the monocyte adhesion to the endothelium caused by AGEs [111]. However, the exact mechanism of metformin action to reduce inflammation processes—directly or indirectly through glycemic normalization—has not been definitively established [112].

It has been found that metformin, through the inhibition of nicotinamide adenine dinucleotide phosphate (NADPH), diminishes ROS production in endothelial cells [113]. The results of the studies conducted by Bakhashab et al. indicate that metformin intensifies the expression of VEGFs responsible for the enhancement of angiogenesis in hypoxia and hyperglycemia conditions [114].

b. NO synthesis

The bioavailability of NO, a potent vasodilator, is one of the key factors in maintaining physiological endothelium properties and function. Metformin has been found to enhance the eNOS-NO pathway through the activation of AMPK in a dose-dependent manner. By this mechanism observed in endothelial cells in vitro, metformin may increase NO-mediated vasodilatation [115].

c. Endothelial senescence and apoptosis

It has been claimed that hyperglycemia could be responsible for the senescence and apoptosis of endothelial cells that eventually lead to the loss of their function. Metformin, by significantly enhancing the expression of SIRT1, has been observed to reduce these processes and enable the endothelium to maintain its properties [116]. SIRT1 has been shown to increase eNOS deacetylation and augment the bioavailability of NO, leading to a reduction of apoptosis and angiogenesis in the endothelium [117].

d. Vascular integrity

Hyperglycemia is one of the factors increasing vascular permeability that finally result in endothelial leakage and the extravasation of monocytes, which is associate with impaired endothelial function. The endothelial glycocalyx, one of the matrix structures, prevents the increase in endothelial permeability. The results from animal model studies have shown that endothelial permeability is inhibited by metformin via AMPK activation, and the glycocalyx barrier is reinforced [118].

**Figure 1:**
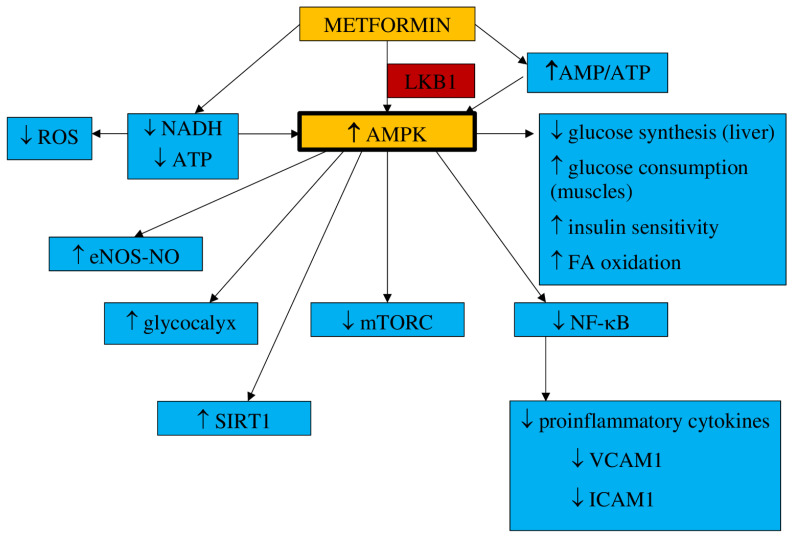
Main mechanisms of metformin action. AMPK—5′-adenosine monophosphate-activated protein kinase, AMP—5′-adenosine monophosphate, ATP—adenosine triphosphate, LKB1—liver kinase B1, NADH—nicotinamide adenine dinucleotide, ROS—reactive oxygen species, eNOS—endothelial nitric oxide synthase, NO—nitric oxide, SIRT1—sirtuin 1, mTORC—mammalian target of rapamycin, FA—fatty acids, NF-κB—nuclear factor kappa B, VCAM1—vascular cell adhesion molecule 1, ICAM1—intracellular adhesion molecule 1, MCP1—monocyte chemoattractant protein 1.

The main mechanisms of metformin action are shown in Figure 1.

### 3.2. Impact on Preeclampsia Pathophysiology

Elevated insulin levels are believed to be exceptionally toxic to trophoblast cells in the first trimester of pregnancy and may be responsible for damage to their DNA, apoptosis, and limiting their survival. Hence, metformin use may prevent these events. These findings also suggest the need to consider screening for insulin resistance before conception to prevent hyperinsulinemia early in pregnancy [119].

It has been suggested that the development of preeclampsia could be related to proinflammatory conditions that lead to the release of free radicals within the placenta and the consecutive oxidative/nitrosative stress [120]. The results of the study conducted by Han et al. revealed that high glucose concentrations had a significant impact on the rise in trophoblast synthesis of proinflammatory cytokines such as IL-1β, IL-6, and IL-8 as well as the synthesis of antiangiogenic factors sFlt-1 and sEnd. They also reduce trophoblast migration. This may indicate the existence of a mechanism linking hyperglycemia to the development of PE and the role of metformin as a potential preventive agent. However, they also observed that metformin limited the glucose-induced inflammatory response moderately without any impact on the antiangiogenic or antimigratory response [121]. This observation has been confirmed by Chiswick et al., who found that women treated with metformin during pregnancy had lower proinflammatory interleukin-6 levels [122]. The metformin impact on inflammation and oxidative stress was examined in numerous animal and in vitro studies. Hu et al. observed that in a rat model of PE induced by lipopolysaccharides (LPS), metformin decreased the LPS-dependent secretion of proinflammatory cytokines such as TNF-α and IL-6 and limited oxidative/nitrative stress by enhancing the activity of superoxide dismutase (SOD). The placental NF-κB signaling pathway, activated by LPS, was suppressed. This resulted in a normalization of blood pressure, reduced proteinuria, improvement in fetal growth, and decreased stillbirth frequency. The authors concluded that metformin is beneficial to the PE-like rat model by protecting placentas from injury; thus, it could be an attractive agent for PE prevention and/or treatment [123]. It has been reported that the decrease in IL-27, TNF-α, and IL-6 expression in vivo (in both preeclamptic rat models and trophoblast cells) was the result of H19 inhibition by metformin in a dose-dependent manner [124]. On the other hand, Correia-Branco et al. showed the adverse influence of metformin on an extravillous trophoblastic cell line, with reducing cell proliferation rates, culture growth, viability, and capacity of migration. Thus, they were of the opinion that the processes involved in placentation could be highly impaired by metformin, with mTORC and PI3K involvement [125].

Metformin acts as an endothelial protective agent via the AMPK activation pathway not only in diabetic patients but also in healthy individuals in a glucose-independent manner [126]. The endothelium dysfunction reported in PE is correlated with an increase in the expression of ICAM1 and VICAM1, which is enhanced by proinflammatory cytokine TNFα [127,128]. Brownfoot et al. revealed that metformin diminished the VCAM1 levels induced by TNF-α in HUVECs (human umbilical vein endothelial cells) [40,129]. An abnormal invasion of the trophoblast leads to ischemia and hypoxia of the placenta and an increase in the concentrations of circulating vasoactive factors. Antiangiogenic factors such as soluble fms-like tyrosine kinase-1 and soluble endoglin cause imbalances in pro- and antiangiogenic factors [130]. The possibility of restoring the balance by suppressing antiangiogenic agents seems attractive as a method of effective prophylaxis of preeclampsia. The results of the study of Brownfoot et al., conducted on endothelial cells, villous cytotrophoblast cells, and preterm preeclamptic placental villous explants, suggest that metformin in a dose-dependent manner decreases the synthesis of sFlt-1 and sENG. It also reversed the endothelial dysfunction observed in preeclampsia. The authors were of the opinion that the metformin effect was likely to be regulated at the mitochondrial level, probably by inhibiting the mitochondrial electron transport chain. In the same study, it has also been observed that the defective angiogenesis caused by sFlt-1 was improved with metformin. The authors of this research concluded that since metformin limited endothelium dysfunction, reinforced vasodilatation, and promoted angiogenesis, it might be useful for the prophylaxis or treatment of preeclampsia [40]. This group of researchers also studied the effect of metformin and esmoprazol belonging to proton pump inhibitors and metformin and sulfasalazine combined on sFlt-1 mRNA expression and sFlt-1 secretion as well as sENG secretion. They have found metformin and esmoprazol to be more effective in decreasing sFlt-1 synthesis, with no additive impact on sENG levels compared to metformin alone [129,131]. The concomitant use of metformin with sulfasalazine resulted in diminished sFlt-1 and sENG release and enhanced VEGF alpha expression in cytotrophoblasts [131].

According to the results of the abovementioned studies, their authors concluded that the combined use of metformin with esmoprazol or sulfasalazine seemed to be more effective in PE prophylaxis and treatment than metformin alone [129,131].

The increase in VEGF release induced by metformin has been revealed in numerous reports. VEGF represents the family of angiogenic factors participating in the development of placental vasculature and appropriate trophoblast invasion and implantation [132]. It has been found that their levels are decreased in preeclampsia [133]. An animal model study showed that metformin enhances VEGF synthesis and, consequently, improves angiogenesis in the placenta [134].

The results of numerous studies have indicated that endothelial function may also be improved as a result of the action of other mechanisms that are modulated by metformin. NO is the leading vasodilator involved in cytotrophoblast invasion, implantation, and providing the development of low-resistance placental blood flow. Since impaired NO bioavailability and signaling have been reported in preeclampsia, a drug that can restore the balance in the NO pathway may be attractive for PE prophylaxis [49]. Metformin has been found to raise NO synthesis through the activation of AMPK, which leads to the activation of eNOS [115].

The results of studies conducted in diabetic patients have indicated that metformin stimulates a marked increase in the number of EPCs and strengthens angiogenic potential by activating the AMPK/eNOS pathway [115,135]. Asadian et al. presented the opposite opinion: they have shown no metformin impact on the number and activity of EPCs [136]. There are also study results that have indicated the adverse effects of metformin on both the number and bioactivity of EPCs. It has been noticed that metformin suppresses the angiogenic capacity of EPCs and their migration [137,138]. The ambiguous conclusions of the research presented above may be the result of different doses of metformin and the small size of the study groups. Hence, it seems that drugs that have a beneficial effect on EPCs might be useful in the prevention or treatment of preeclampsia.

Endoplasmic reticulum stress is believed to be involved in the pathogenesis of preeclampsia by promoting the release of proinflammatory cytokines, antiangiogenic factors, and trophoblastic apoptotic debris [57,58,59]. The results of the study of Suzuki et al., conducted in trophoblast-like cells, indicated that metformin, by limiting ERS, restored normal levels of PIGF, which might justify its use in the prevention of PE [139].

Placentation, which is impaired in preeclampsia, requires extracellular matrix degradation with the involvement of metalloproteinases [61]. Wang et al., conducting a study on the effect of metformin on PE-like animal models, have found that it improved vascularization and contributed to an increase in the concentration of MMP-2 and VEGF in preeclamptic placental tissue [134].

Additionally, metformin, by inducing SIRT1 expression, is believed to significantly increase cell viability, decrease cell apoptosis, and reduce the release of proinflammatory cytokines, which allow the maintenance of physiological endothelium function [72]. There are few reports on SIRT1 in preeclampsia, and data on the metformin effect on SIRT1 in preeclampsia are, so far, unavailable.

**Figure 2:**
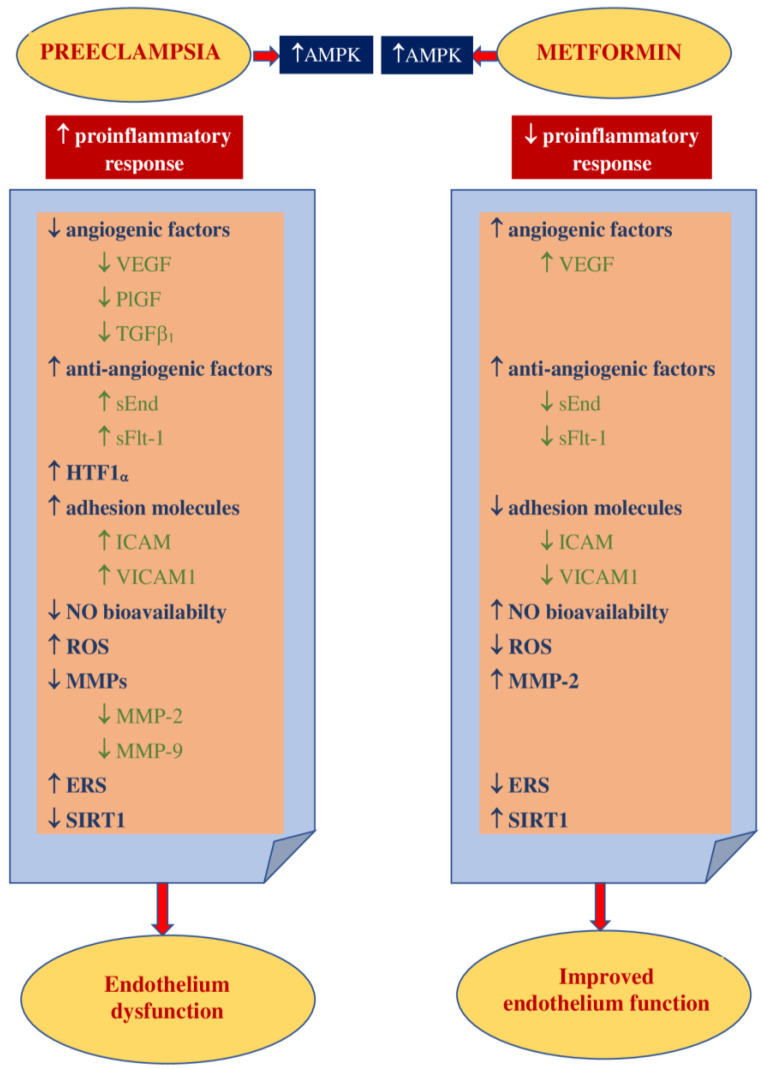
Theoretical basis for the use of metformin in PE prevention. AMPK—5’ adenosine monophosphate-activated protein kinase, VEGF—vascular endothelial growth factor, PlGF—placental growth factor, TGF β_1_—transforming growth factor-β_1_, sEnd—soluble endoglin, sFlt-1—fms-like tyrosine kinase-1, HTF1 α_—_hypoxia-inducible factor 1α, ICAM1—intracellular cell adhesion molecule 1, VICAM1—vascular cell adhesion molecule 1, NO—nitric oxide, ROS—reactive oxygen species, MMPs—matrix metalloproteinases, MMP-2—matrix metalloproteinase-2, MMP-9—matrix metalloproteinase-9, ERS—endoplasmic reticulum stress, EPCs—endothelial progenitor cells, SIRT1—sirtuin1.

Figure 2 shows the main theoretical basis for the use of metformin in PE prophylaxis.

## 4. Metformin in Preventing Hypertensive Disorders of Pregnancy

Due to the effect of metformin, far beyond the impact on carbohydrate metabolism, it becomes an attractive drug for the prevention of hypertensive disorders of pregnancy.

The studies published so far have focused mainly on its effects on pregnancy outcomes and the fetus and child and were conducted primarily in women with gestational diabetes (GDM), polycystic ovary syndrome (PCOS), and obesity. The main objective of these studies was not to assess the effect of metformin on the development of hypertensive complications in pregnancy.

This chapter presents the results of randomized controlled trials (RCTs) and meta-analyses that have been published over the past 10 years. Electronic databases Pubmed and MEDLINE were searched using keywords such as metformin and pregnancy. Only articles available in English were taken into account. Of the 110 published RCTs, only 10 provided information on the effect of metformin on preeclampsia and/or pregnancy-induced hypertension incidences, and 11 out of 74 meta-analyses did.

The results of the selected randomized controlled trials that have been published within the last 10 years and provide information on metformin influence on the frequency of preeclampsia are presented in Table 1. Some analyses have also taken into account the incidence of pregnancy-induced hypertension or gestational hypertension; these terms were used interchangeably for gestational hypertension. In none of the following work metformin impact on the incidence of hypertensive complications of pregnancy was the primary outcome.

The results of the above studies are inconclusive, and attention should be paid to the significant differences in their protocols. They were carried out on obese pregnant women (BMI > 30 or > 35 kg/m^2^) or in patients with GDM, DMt2, or polycystic ovary syndrome (PCOS). The most common comparison was the effect of metformin on the pregnancy course with the effect of insulin. Only studies in obese pregnant women or women with PCOS compared the impact of metformin to placebo or no treatment [122,146,147,148]. The dose of metformin used was also not uniform: treatment usually started with a dose of 500 mg, which was increased at different intervals to the maximum dose of 3000 mg [147], although in most studies, the maximum dose was 2500 mg [122,143,145].

Metformin administration was started at different gestational ages, from the 6th week up to the 36th week, and continued to childbirth; this may be a key issue when it comes to preeclampsia prevention [141,142,143]. Aspirin, which is recommended for PE prophylaxis, should be introduced up to a maximum of the 12–16th week (depending on the recommendations), that is, before the end of the trophoblast invasion [6,7,8].

In obese pregnant women, a significant reduction in PE frequency was observed in two studies [146,147] without such effect as in the research carried out by Chiswick et al. [122]. There was no reduction in the incidence of PIH in obese pregnant women under the influence of metformin, although, in all studies, its administration was started before the 20th week [122,146,147].

In women with GDM, metformin administration was not unequivocally effective in the prevention of PE. Brink et al., offering metformin at the dose of 500–1000 mg from the 14th week, reported a significant decrease in PE incidence compared to insulin treatment [140]. Ainuddin et al. also showed a significant reduction in PE frequency, but the results of these studies should be treated with extreme caution as metformin administration was started between 20 and 34 weeks [141]. The other authors have not demonstrated its beneficial effect on the prevention of PE or PIH [143,144].

For pregnant women with DMt2, however, one study demonstrated the more important role of metformin in preventing PIH compared to PE [145], while Feig et al. did not observe similar effects [144]. Differences in the results of both studies may be the effect of different gestational ages at which the metformin treatment was offered: approximately 10 weeks vs. 6–22 weeks [144,145].

In women with PCOS, metformin was not effective in preventing either PE or PIH, although it was offered since the first trimester [148].

Table 2 presents the results of the meta-analyses that have been published in the last 10 years and provide information on metformin influence on the frequency of PE or PIH or both. It should be noted that only in the meta-analysis of Nascimiento et al., preeclampsia or gestational hypertensive complications were the primary outcomes [149].

Additionally, the results of the presented meta-analyses do not allow us to draw clear conclusions on the effectiveness of metformin in the prevention of hypertensive complications of pregnancy. As in the case of randomized studies and the meta-analyses presented above, the significant differences between the groups assessed should be highlighted. The studied groups were heterogeneous: some analyses included both pregnant women with obesity and women with GDM or PCOS [149] or obesity and GDM [150]; others evaluated groups only with obesity [159] or GDM [151,152,153,154,155,156] or PCOS [158,159]. The presented meta-analyses did not assess studies on metformin influence on pregnancies complicated by diabetes mellitus t.2.

The most common comparison was metformin effect on the pregnancy course and outcomes with the effect of insulin (GDM patients) [151,152,153,154,155,156]. Only meta-analyses on obese pregnant women or patients with PCOS have compared the effects of metformin with placebo or no treatment [157,158,159].

The results in the obesity group have been inconclusive. Dodd et al. did not recorde the efficacy of metformin in reducing PIH [157], while Nascimiento et al. showed rarer PE occurrence [150]. In the group of women with GDM, there was a significant effect of metformin on the reduction of the risk of PIH compared to insulin [149,151,153,154,156], while no such effect was noted on the reduction of PE risk [149,153,155].

The conclusions on the effectiveness of metformin compared to placebo or no treatment in women with PCOS on the incidence of HDPs were even more ambiguous. Feng et al. did not observe the effect of its use on PE frequency [158], Nascimiento et al. revealed a significant reduction in PIH frequency but not PE [149], while the results of the meta-analysis of Zheng et al. indicated a lower risk of developing PE when metformin was administered [159].

In addition, the research of Stridsklev et al., conducted in the group of women with PCOS, showed that metformin compared to placebo did not change the PI value and, thus, did not affect blood flow through uterine arteries at 19 ± 1 weeks of pregnancy. The PI value correlated positively with PE and hypertension in pregnancy and gestational diabetes [160]. However, some authors have questioned the usefulness of the PI assessment in predicting preeclampsia. The PREDO study, evaluating the efficacy of aspirin or placebo in women with abnormal PI in the prevention of PE, did not show a significant difference [161].

Based on the above data, it can be concluded that the most promising results of metformin use in the context of hypertension prophylaxis in pregnancy were demonstrated in the group of women with GDM. Since GDM, in the vast majority of cases, is diagnosed after 20 weeks, that is, after trophoblast implantation is complete, consideration should be given to the more significant impact of metformin on the other elements responsible for the development of hypertensive disorders of pregnancy.

The results of a study by Martis et al., who compared the effect of insulin on the course and outcomes of pregnancy in women with GDM with oral hypoglycemic drugs, including metformin, seem to be intriguing. They revealed that neither insulin nor oral medicines affected the risk of PE development, but insulin significantly increased the incidence of hypertensive disease of pregnancy [162].

## 5. Conclusions

At the moment, only one agent—aspirin—is recommended for the prevention and development of preeclampsia. Studies are ongoing on the efficacy of other active medical substances and drugs that, due to their mechanism of action and properties, could be offered for the prevention and/or treatment of HDPs. One such drug is metformin, which has much wider than just hypoglycemic properties, long used to treat diabetes. Based on the results of studies explaining its mechanism of action and the reasons and pathophysiology of the development of preeclampsia, it might be postulated that an anti-inflammatory effect of metformin and its beneficial influence on the endothelium constitutes its potential use in the prevention and/or treatment of PE and PIH. In addition, it is a safe drug, long used for the treatment of gestational diabetes. Unfortunately, the results of randomized controlled trials and meta-analyses are ambiguous; they assessed the effect of metformin on the incidence of PE and PIH while treating metabolic diseases (GDM, PCOS, DMt.2, or obesity) that are recognized risk factors for HDP development. It is possible that these pregnant women are the group that would benefit the most from the prophylactic use of metformin.

In the light of available data, however, it seems that, at present, more questions on such metformin applications arise. The most important are as follows: (1) whether more detailed algorithms of PE prediction (angiogenic and antiangiogenic factors, PI value in uterine arteries) before metformin is included should be considered; (2) when and what dose of metformin should be applied; (3) what influence of the concomitant metformin and aspirin administration would have on the incidence of preeclampsia; (4) whether pregnant women with other chronic conditions such as chronic hypertension and autoimmunological diseases will benefit from the prophylactic use of metformin.

These questions may set new directions for research on the use of an old drug with new properties in the prevention of hypertensive complications of pregnancy.

## Figures and Tables

**Table 1:** Selected randomized placebo-controlled trials on metformin influence on PE and PIH incidence.

Studied Group	Size of Groups	Metformin Dose	GA at Entry to the Study	PIH and PE	Authors
GDM high risk	SG: metformin—24 CG:no treatment—25	500–1000 mg	14th week	**PE** SG: 0% (0) CG: 8.7% (2) *p* = 0.049	Brink et al., 2018 [140]
GDM	SG: metformin—43 CG:insulin alone—57	500–2500 mg	20th–36th week	**PIH** SG: 18.6% (8) CG: 24% (18) NS **PE** SG: 0% (0) CG: 8% (6) *p* = 0.05	Ainuddin et al., 2014 [141]
GDM	SG: metformin 110 CG insulin 107	500–2000 mg	22nd–34th week	**PIH** SG: 1.8% (2) CG: 3.7% (4) *p* = 0.42, RR 0.5 95% CI 0.1–2.7 **PE** SG: 4.6% (5) CG: 9.4% (10) *p* = 0.19, RR 0.5 95% CI 0.2–1.4	Tertti et al., 2013 [142]
GDM	SG metformin: 86 CG insulin: 80	1000–2500 mg	20th–34th week	**PIH** SG: 5% (4) CG 13.8% (11) *p* = 0.058, RR 0.4 95% CI 0.1–1.1 **PE** SG: 6.3% (5) CG: 8.8% (7) *p* = 0.548, RR 0.7 95% CI 0.2–2.22	Niromanesh et al., 2012 [143]
DMt.2	SG: metformin—233 CG: insulin—240	2000 mg	6th–22th week	**PIH** SG: 5% (13) CG 6% (15) *p* = 0.82, RR 0.92 95% CI 0.46–1.8. **PE** SG: 15% (37) CG 12% (30) *p* = 0.29, RR 1.27 95% CI 0.82–1.92 Chronic HT ST: 8% (20) CG: 9% (22) *p* = 0.68, RR 0.89 95% CI 0.51–1.56	Feig et al., 2020 [144]
DMt.2	SG: metformin alone—16 CG: insulin alone 100	500–2500 mg	about 10th week	**PIH** SG: 6.2% (1) CG: 36% (36) *p=* 0.020 **PE** SG: 25% (4) CG: 17% (17) *p* = 0.084	Ainuddin et al., 2015 [145]
Obesity	SG: metformin—171 CG: placebo—186	1000 mg	<20th week	**PE** SG: 3.5% (6) CG: 4.8% (9) *p* = 0.01, RR 0.17 95% CI 0.10–1.41	Nascimento et al., 2020 [146]
Obesity (35 kg/m2)	SG: metformin—202 CG: placebo—198	1000–3000 mg	12th–18th week	**PIH** SG: 6.4% (13) CG: 6.7% (13) *p =* 0.93, RR 0.96 95% CI 0.43–2.13 **PE** SG: 3% (6) CG: 11.3% (13) *p* = 0.001, RR 0.24 95% CI 0.10–0.61	Syngelaki et al., 2016 [147]
Obesity (BMI > 30 kg/m2)	SG: metformin—221 CG: placebo—222	500–2500 mg	12th–16th week	**PIH** SG: 10% (21) CG 6% (14) *p=* 0.22, RR 1.56 95% CI 0.77–3.15. **PE** SG: 3% (7) CG 1% (3) *p* *=* 0.21, RR 2.39 95% 0.61–9.36	Chiswick et al., 2015 [122]
PCOS	SG: metformin—238 CG: placebo—240	1000–2000 mg	in the 1st trimester	**PE** SG: 3% (8) CG: 7% (17) *p =* 0.10, RR 0.46 95% CI 0.17–1.15	Løvvik et al., 2019 [148]

GDM—gestation diabetes mellitus. DMt2—diabetes mellitus t.2. BMI—body mass index. PCOS—polycystic ovary syndrome. SG—study group. CG—control group. PIH—pregnancy-induced hypertension. PE—preeclampsia. HT—hypertension. *p*—statistical significance. RR—relative risk. CI—confidence interval.

**Table 2:** Selected meta-analyses on metformin influence on PE and PIH risk.

Studied Group	Comparison	Number of Participants	Metformin Impact on PIH/PE	Authors
GDM	metformin vs. insulin	1260	**PIH** RR 0.56 95% CI 0.37–0.85 **PE** RR 0.83 95% CI 0.60–1.14 **PE** RR 0.74 95% CI 0.09–6.28	Kalafat et al., 2018 [150]
Obesity	metformin vs. placebo	840		
GDM	Metformin vs. insulin	2165	↓**PIH** RR 0.56 95% CI 0.37–0.85	Butalia et al., 2017 [151]
GDM	metformin vs. insulin	1556	↓**HDPs** RR 0.82 95% CI 0.67–1.0	Feng et al., 2017 [152]
GDM	metformin vs. insulin	1110	↓**PIH** RR 0.53 95% CI 0.31–0.90 **PE** RR 0.81 95% CI 0.55–1.17,	Li et al., 2015 [153]
		1634		
GDM	metformin vs. insulin	1110	↓**PIH** RR 0.55 95% CI 0.31–0.91 **PE** RR 0.84 95% CI 0.57–1.23	Poolsup et al., 2014 [154]
		1299		
GDM	metformin vs. insulin	1712	**PE** RR = 0.82 95% CI 0.56–1.2	Zhu et al., 2014 [155]
GDM	metformin vs. insulin	1110	↓**PIH** RR 0.52 95%CI 0.30–0.90	Gui et al., 2013 [156]
Obesity	metformin vs. no-treatment	840 614 308	**PIH** (obesity) RR 1.24 95% CI 0.76–2.02 ↓**PE** (obesity) RR 0.51 95% CI 0.26–0.98 ↓**PIH** (PCOS) RR 0.37 95% CI 0.25–0.57 **PE** (PCOS) RR 1.96 95% CI 0.81–4.77 ↓**PIH** (GDM) RR 0.53 95% CI 0.31–0.90 **PE** (GDM) RR 0.70 95% CI 0.45–1.10	Nascimento et al., 2018 [149]
PCOS				
GDM	metformin vs. insulin	1120 1120		
Obesity	metformin vs. no-treatment or placebo	1034	**PIH** RR 1.02 95% CI 0.54–1.94 **PE** RR 0.74 95% CI 0.09–6.28	Dodd et al., 2018 [157]
PCOS	metformin vs. no-treatment or placebo	929	**PE** RR 0.92 95% CI 0.28–3.00	Feng et al., 2015 [158]
PCOS	metformin vs. no-treatment or placebo	878	↓**PE** RR 0.53 95% CI 0.30–0.95	Zheng et al., 2013 [159]

GDM—gestation diabetes mellitus. PCOS—polycystic ovary syndrome. PIH—pregnancy-induced hypertension. PE—preeclampsia. HT—hypertension. RR—relative risk. CI—confidence interval.

## Data Availability

MDPI Research Data Policies.
